# Nurses’ Clinical Practice in Nursing Homes: Depressive Symptoms and Fall Risk Assessment

**DOI:** 10.3390/geriatrics9060158

**Published:** 2024-12-09

**Authors:** Alcina Matos Queirós, Armin von Gunten, Maria Manuela Martins, Henk Verloo

**Affiliations:** 1 Institute of Biomedical Sciences Abel Salazar, University of Porto, 4050-313 Porto, Portugal; 2 Department of Health and Social Welfare, 1018 Lausanne, Switzerland; 3 Service of Old Age Psychiatry, Lausanne University Hospital and University of Lausanne, 1008 Prilly, Switzerland; 4 School of Nursing Sciences, University of Applied Sciences and Arts Western Switzerland, 1950 Sion, Switzerland

**Keywords:** depression, fall risk, assessment, nurses, nurses reported practices, nurses perceptions, nursing homes

## Abstract

Background: Depression and falls are highly prevalent, interrelated concerns for nursing home (NH) residents. Relationships between depression and falls should guide nurses towards developing evidence-based practices for assessing these conditions together. This study aimed to ascertain NH nurses’ clinical practices and perceptions regarding the assessment of depression and fall risk. Methods: This study was an exploratory descriptive study on the reported practices and perceptions from NH nurses in the canton of Vaud, Western Switzerland. Statistical analyses included descriptive statistics, nonparametric tests and a content analysis of responses to open-ended questions. Results: The mean age of our 116 responding nurses was 44.6 years old (SD = 11.3), 99 were women and their mean work experience in NHs was 13.1 years (SD = 9.2). The reporting showed that 88.8% of nurses relied on mood observation for assessing depression and 88.8% relied on the history of falls to identify fall risk. Only 75.9% and 61.2% of nurses used validated scales to detect depression and fall risk, respectively. Additionally, 56.9% of participants considered depression to be a significant factor in fall risk. Conclusion: Validated tools to assess depression and fall risk in NHs should be used more widely. Health policies must support and enhance NH nurses’ training and skills.

## 1. Introduction

### Background

Worldwide, people are living longer, healthier lives. This global demographic transition is a serious issue for Switzerland, where the number of people aged 65 or older is expected to reach 2,700,000 by 2045 [1]. For the canton of Vaud in Western Switzerland, the number of older adults (OAs) aged 65 and over is expected to rise to 248,000 by 2050, compared to less than 135,000 at the end of 2020 [2], and the number of older adults aged 80 and over will likely more than double by 2050, from 38,400 in 2020 to 89,000 [2].

Even though life expectancy without severe or mild disabilities is trending upward across Switzerland, the need for long-term care is increasing rapidly due to the population’s growing longevity [3,4]. For a fraction of OAs, admission to an NH becomes essential to ensure that they can continue to live in safety and maintain a satisfactory quality of life [3,5,6]. Chronic multimorbidity is highly prevalent among Switzerland’s NH residents: 86% have several diagnosed illnesses, with 78% having a diagnosed somatic pathology and 69% having a mental one [7]. When institutionalisation in an NH is unavoidable, the associated somatic, psychological and social changes create a period of risk during which mental health problems may exacerbate [8]. A recent systematic review on this subject reported evidence of rates of depression between 14% and 22% of OAs living in the community, compared to between 32% and 63% of those who are institutionalised [8]. Other studies have described the prevalence of depression among nursing home residents at between 32% [9] and 48% [10] when validated tools were used to assess mental health difficulties. These findings may corroborate the idea that depression among NH residents often goes unrecognised and may be a neglected part of the ageing process [11]. Few studies have investigated depression among Switzerland’s NH residents. A 2012 report on institutionalised OAs’ health revealed that 28% of them presented with a diagnosis of depression, and 34% without a diagnosis of depression presented with depressive symptoms [7].

Another common issue for NH residents is falls. Falls are considered the most common cause of injuries among OAs in general [12]. In NH settings, the prevalence of falls has ranged from 13.0% [13] to 92.5% [14]. A study conducted among Swiss NH residents by Hoedl et al. (2022) found that 15.8% of residents experienced their first fall after being admitted to the institution. Falls have serious negative consequences on NH residents’ health, being responsible for considerable dependence on staff, hospitalisations or even early death [15]. More than 20% of OAs’ falls in the United States are estimated to require a hospital visit [16]. Over one year, 39% of Switzerland’s NH residents had a fall and almost a quarter fell several times [7]. Falls increase health costs due to the higher probability of needing hospital care, hospitalisation or surgery [17,18].

Depression and falls have a complex, bidirectional relationship [19]. Kvelde et al.’s (2013) systematic review and meta-analysis reported that individuals with higher levels of depressive symptoms at baseline were more likely to experience falls during the follow-up period (OR = 1.46, 95% confidence interval (CI) = 1.27–1.67, *p* < 0.001, I(2) = 77.2%). Similarly, Shao et al. (2023) identified depression as a key psychological factor associated with falls in NHs, with an odds ratio of 1.68 (95%CI = 1.05–2.97) [20]. Additionally, studies within their analysis also reported a risk ratio (RR) for depression of 1.44, further underscoring the relationship between depressive symptoms and fall risk among NH residents [20]. Collectively, these findings emphasise the importance of addressing depression as a critical factor in fall prevention strategies for older adults in NH settings.

NH residents often present with complex chronic health conditions, and healthcare systems and professionals must be prepared to respond to their specific needs. Nurses are pivotal in the comprehensive geriatric assessment process and are often expected to take leading roles and coordination responsibilities in caring for older adults [21]. Their intimate, continuous interactions with NH residents often position them as the first professionals to observe changes in residents’ mental and physical health [21].

Nurses’ specific role in comprehensive geriatric assessment remains poorly defined, and the existing literature fails to address it thoroughly [21]. Despite the well-documented relationship between depression and falls among NH residents, to the best of our knowledge, little is known about how these issues are assessed in Switzerland’s NHs, particularly from the perspective of nurses. This gap in the literature demonstrates the importance of exploring nurses’ practices and perspectives in order to improve the quality of NH care. Gaining insight into how nurses evaluate depression and fall risk will allow us to better support their critical role in early detection and intervention, ultimately contributing to safer, more responsive care for NH residents.

Given their central role in patient care within NHs, nurses are crucial in conducting assessments to optimise quality personalised care.

Therefore, this study’s main objectives were as follows: (i) analyse nurses’ perceptions and reported practices for assessing depressive symptoms and fall risk; (ii) explore relationships between participating nurses’ reported practices regarding depression and fall risk assessment and their sociodemographic and professional characteristics; (iii) explore nurses’ perceptions about the risk factors for falls; and (iv) explore which factors were predictive of nurses using validated scales for assessing depressive symptoms and fall risk.

Nurses have a fundamental role and responsibility to play in identifying depression, but the role of formally diagnosing depression belongs to a physician. In this study, however, the terms ‘depression’ and ‘depressive symptoms’ are used synonymously to facilitate reading.

## 2. Materials and Methods

### 2.1. Study Design

We designed a cross-sectional descriptive study to collect the opinions and clinical practices of nurses working in eligible NHs in the canton of Vaud in the French-speaking part of Western Switzerland. This study’s components are reported based on the STROBE reporting guidelines [22].

### 2.2. Study Framework

**Figure 1:**
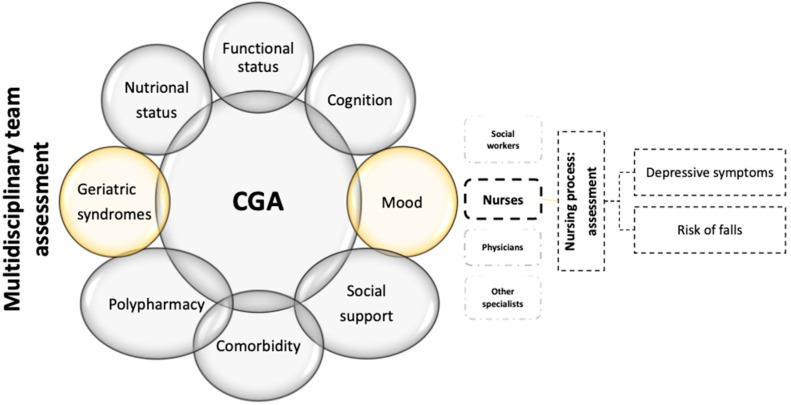
The survey variables explored by the comprehensive geriatric assessment and the Nursing Process approach—inspired by Spirgiene and Brent [21].

The present study was based on components of the comprehensive geriatric assessment framework and the Nursing Process approach. Several countries promote the comprehensive geriatric assessment framework as a suitable multidimensional, multidisciplinary assessment process that can guide professionals and encourage best practices in identifying OAs’ medical, social and functional needs [23]. Although there is no universal consensus on which components to use, key interdisciplinary elements include functional status, cognition, mood, social support, nutrition, comorbidities, polypharmacy and geriatric syndromes [23]. The comprehensive geriatric assessment recommends using validated, geriatric-specific scales to identify health issues, using personalised care goals and planning. It also assesses NH residents’ social and environmental circumstances, focusing on social support, networks and the safety of their living environments [24]. For nurses, the Nursing Process is a structured approach that employs scientific reasoning, problem-solving and critical thinking to guide their delivery of effective patient care. Widely recognised in nursing practice, this approach is scientific and dynamic, and involves five key phases—assessment, diagnosis, planning, implementation and evaluation—aimed at ensuring high standards of care. NH nurses play a key role in assessing residents’ individual needs through their close, daily interactions [25]. In this study, the authors focused on the depressive symptoms and fall risk identified by nurses’ daily clinical assessment practices and perceptions (Figure 1).

### 2.3. Population, Setting and Sample

The population of interest was front-line nurses working in the canton of Vaud’s eligible NHs. These NHs provide care in two main clinical specialities: geriatrics and old age psychiatry. The latter are distinguished by the care they provide to individuals with cognitive, affective or psychotic disorders.

A total of 110 NHs in the canton were identified as eligible, and they were all invited to participate in this study. All the canton’s NHs are members of local NH federations. The study was presented to these local federations, all of which actively supported the dissemination of our questionnaire by encouraging participation among their member NHs.

Our study applied a convenience sampling method to ensure a broad range of participants. All the NH management teams in the canton were asked to distribute the self-reporting questionnaire to at least one of their front-line nurses. The inclusion criterion for each NH was to have at least one participating nurse, with no exclusion criteria regarding their age or level of work experience.

### 2.4. Survey Instrument

Because they were unable to identify any psychometrically validated instruments suitable for addressing the present study’s aims, the authors designed an ad hoc self-reporting questionnaire using information found in a literature review and the authors’ clinical and empirical experiences (Supplementary Materials File S1). The first section consisted of seven closed and two open questions about participants’ sociodemographic and professional information (age, sex, professional experience, professional experience in NHs and advanced training) and their NH’s clinical speciality (geriatric care, old age psychiatry or both). The second section collected information about nurses’ empirical experiences and daily clinical practices regarding the assessment of depressive symptoms and fall risk. This section included six closed and two open questions to obtain exhaustive information on this central theme. The third section comprised two questions seeking to identify risk factors for falls in NHs from the participants’ perspectives and to assess the extent to which they agreed that depression increases fall risk. To ensure that instructions were clear and questions were understandable and well structured, pilot tests on the questionnaire were conducted with three nurses—one with experience in conducting survey studies and two with experience working in NHs. Based on their feedback, minor wording corrections were made, and the suggested time required to complete the survey was adapted to 15–20 min.

### 2.5. Data Collection

The authors conducted the survey online, using Google Forms—a method chosen for both sustainability and practical reasons. Multiple strategies were employed to maximise response rates and reach a maximum number of eligible participants.

The questionnaire was emailed to the management teams of the 110 eligible NHs across the canton. This was accompanied by comprehensive information about this study and a Free and Informed Consent Form. Participants had the choice of completing the questionnaire online, directly through Google Forms, or, if they so preferred, they had the option of returning a paper printout of their responses by post.

Two email reminders were sent out to the NH management teams during the six-week data collection period, between January and March 2024, to encourage them to complete the questionnaire and maximise participation. Reminders were sent out two weeks and four weeks following the initial email.

Additionally, the local NH federations actively reinforced our reminders in their newsletters to their members, further encouraging participation.

### 2.6. Data Analysis

After inspecting the Excel^®^ spreadsheet dataset for extraction errors, missing values and data consistency, it was imported into Statistical Package for the Social Sciences (SPSS) software, version 29.0 (IBM-SPSS Inc, Chicago, IL, USA), for analysis. Missing values were analysed based on best practices for cross-sectional datasets. Specifically, three missing observations related to age were identified. These missing values were imputed using the mean age of the sample to ensure consistency and minimise bias in the analysis.

We conducted a descriptive analysis that calculated means and standard deviations for continuous and ordinal variables and frequencies for categorical variables. This analysis focused on participants’ sociodemographic and professional characteristics and nurses’ perceptions and reported on their practices regarding the assessment of depressive symptoms and fall risk.

Per this study’s purpose, advanced training and validated scale variables were recoded as dichotomous variables for the planned statistical analysis. Nonparametric tests were performed for variables with non-normal distributions to compare NHs and nurses’ characteristics. The chi-squared test was used to compare nurses’ sociodemographic and professional characteristics and clinical practices when assessing depressive symptoms and fall risk using validated scales. Spearman’s rank test was performed to explore correlations between nurses’ advanced training in geriatrics or old age psychiatry and their use of validated scales to assess depressive symptoms and fall risk. Multivariate logistic regressions were calculated to explore the relationships between participants’ sociodemographic and professional characteristics and their use of validated scales to assess depressive symptoms and fall risk. The statistical significance level was set at *p* < 0.05 for all the analyses. Using NVivo 13 software, a content analysis of the answers to the open-ended questions was conducted to find emergent themes.

### 2.7. Ethical Considerations

Since our study only collected nurses’ perceptions and reported practices and did not collect any data on the residents’ health, the canton of Vaud’s Department of Health and Social Welfare and its regional NH federations approved this study. Participating nurses received complete information about this study and signed a Free and Informed Consent Form. By returning the questionnaire to the investigator, respondents gave their tacit consent to voluntarily participate in this study and agreed to its conditions. All the information collected in the survey was anonymous.

## 3. Results

### 3.1. Participant Response Rates, NHs’ Principal Speciality and Cantonal Health Regions

A total of one hundred and sixteen nurses responded to the questionnaire, with one hundred and ten (94.8%) answering online and six (5.2%) returning their completed questionnaires by post.

Most nurses (n = 70; 60.3%) who responded to the questionnaire were employed in an NH specialising in both geriatric and old age psychiatric care, 25 (21.6%) worked in an NH specialising in geriatrics alone, and 21 (18.1%) worked in an NH specialising in old age psychiatry.

### 3.2. Participants’ Sociodemographic and Professional Characteristics

Most participants were women (n = 99, 85.3%), and the mean participant age was 44.6 ± 11.3 years old. On average, participants had worked as nurses for 18.4 ± 10.4 years, with 13.1 ± 9.2 years in an NH.

Forty-four (37.9%) nurses completed advanced training in geriatrics and fifty-one (44.0%) completed it in old age psychiatry: fifteen (12.9%) nurses had a Diploma of Advanced Studies in Ageing Population Health, ten (8.6%) had a Certificate of Advanced Studies in Palliative Care, four (3.4%) had a Certificate of Advanced Studies in Clinical Care and one (0.9%) had a Master’s degree in Nursing Science. Twenty-seven (23.3%) nurses had a Certificate of Advanced Studies in Old Age Psychiatry and eight (7.8%) had completed the canton of Vaud’s ‘Old Age Psychiatry Caregiver’ programme. The remaining 24 (17.2%) nurses with complementary training in geriatrics and 12 (10.3%) with complementary training in old age psychiatry had attended a variety of advanced training programmes in those domains. Table 1 gives more detailed information.

### 3.3. Clinical Practices for Assessing Depressive Symptoms and Fall Risk

Eighty-four (72.4%) and eighty-seven (75.0%) nurses reported basing their clinical nursing reasoning on experience and intuition when assessing depressive symptoms and fall risk, respectively. Sixty-four (55.2%) nurses reported basing their clinical reasoning on a validated scale to evaluate depressive symptoms, while forty-three (45.7%) reported doing so to assess fall risk.

Most respondents (n = 103; 88.8%) reported that they evaluated NH residents’ depressive symptoms by observing mood, particularly signs of social withdrawal or changes in readiness to participate in daily activities. Similarly, 111 (95.7%) nurses reported observing residents’ gait patterns to determine their fall risk, and almost all (n = 103; 88.8%) collected information on residents’ histories of falls over the past year to evaluate that risk. Finally, 65 (56.0%) respondents mentioned referring patients to physicians to complete depression assessments, but only 31 (26.7%) referred them to physicians to evaluate fall risk.

In the open-ended questions, 44 nurses reported that they had discussions with residents and their families to explore depressive symptoms, and 21 specifically mentioned individual clinical interviews with residents at risk of mood disorders. Nine nurses reported calling in mobile teams of old age psychiatry experts who supported them in assessing residents’ depressive symptoms.

Twenty nurses stated that they performed complementary somatic evaluations for depressive symptoms and fall risk (looking particularly at vital signs, sleep and weight). Sixteen nurses reported engaging in multidisciplinary clinical discussions to assess depressive symptoms with their NH colleagues and with mobile teams. Additionally, six nurses mentioned participating in similar discussions for assessing fall risk.

Finally, nurses mentioned how the survey had drawn their attention to the often overlooked issue of depression among NH residents, which is less studied than dementia.

### 3.4. Using Validated Scales to Assess Depressive Symptoms and Fall Risk

Eighty-eight (75.9%) nurses reported using at least one validated scale for assessing depressive symptoms, most commonly the 15-item Geriatric Depression Scale, used by 53 (45.7%) respondents; the Cornell Scale for Depression in Dementia, used by 38 (32.8%); and the 4-item Geriatric Depression Scale, used by 30 (25.9%). A further eight (6.9%) nurses used the 30-item Geriatric Depression Scale, three (2.6%) used the Edmonton Symptom Assessment System, two (1.7%) used Beck’s Depression Inventory, and one (0.9%) used the Hamilton Depression Rating Scale.

Seventy-one (61.2%) nurses reported using a scale to assess fall risk, with thirty-one (26.7%) relying on the Morse Fall Scale, 26 (22.4%) using the Tinetti Performance-Oriented Mobility Assessment and six (5.2%) using the STRATIFY scale. Furthermore, 18 (15.5%) respondents used the Timed Up and Go test, and 14 (12.1%) used the 6-Metre Walk Test.

Each participating nurse was also asked about their perception of how frequently their peers used validated scales in their daily practice to assess the presence of depressive symptoms among NH residents. Forty-four (37.9%) thought this occurred frequently, very often or always. Fifty-four (46.6%) believed that nurses frequently, very often or always used a validated scale to assess fall risk (see Table 2 for details).

### 3.5. Associations Between Nurses’ Sociodemographic and Professional Characteristics and Their Use of Validated Scales for Assessing Depressive Symptoms and Fall Risk

Nurses who had advanced training in geriatrics were statistically significantly more likely to have used validated scales to assess depressive symptoms (*p* = 0.003) and fall risk (*p* = 0.047) than those who did not have that training. Similarly, nurses with advanced training in old age psychiatry were statistically significantly more likely to have used validated scales to assess depressive symptoms (*p* = 0.047) than those without that training. Although a higher percentage of nurses with advanced training in old age psychiatry used validated scales for assessing fall risk, the difference with less well-trained nurses was not statistically significant (*p* = 0.066). Older nurses (aged ≥35) were statistically significantly more likely (*p* = 0.014) to have used validated scales for assessing depressive symptoms more frequently than younger nurses (aged <35). No significant difference was found between the different age groups of nurses in their use of validated scales for assessing fall risk in their daily practice (*p* = 0.071).

Finally, a statistically significant difference was found in the use of validated scales for assessing fall risk (*p* = 0.034) depending on the NH’s principal speciality. The percentage of scale usage was notably higher among respondents working in NHs combining the geriatrics and old age psychiatry specialities than among those working in NHs specialising in old age psychiatry or geriatrics alone.

No significant differences were found in the use of validated scales for assessing depressive symptoms and fall risk based on nurses’ years of professional experience (all *p* > 0.01) (see Supplementary Materials File S2 for details).

### 3.6. Correlations Between Nurses’ Sociodemographic and Professional Characteristics and Their Clinical Practices Using Validated Scales for Assessing Depressive Symptoms and Fall Risk

A low-to-moderate significant positive correlation was found between having advanced training in geriatrics and using a validated scale for assessing depressive symptoms (Rs = 0.275; *p* < 0.01). There was also a low positive correlation between having advanced training in geriatrics and using a validated scale for assessing fall risk (Rs = 0.185; *p* < 0.05). Similarly, a low-to-moderate significant positive correlation was found between having advanced training in old age psychiatry and using a validated scale for assessing depressive symptoms (Rs = 0.256; *p* < 0.01). The association between having training in old age psychiatry and using a validated scale for assessing the risk of falls was also positive but less pronounced, with marginal significance (Rs = 0.171; *p* < 0.05). See Table 3 for details.

### 3.7. Multivariate Logistic Regressions

Multivariate logistic regressions were calculated to assess whether the participants’ professional characteristics predicted their use of validated scales to assess depressive symptoms and fall risk when adjusted for age and sex. The results in Table 4 and Table 5 indicate that certain professional characteristics significantly predicted the likelihood of using validated scales for both assessments.

Table 4 presents beta coefficients (B) and corresponding odds ratios (ORs), suggesting that advanced training in geriatrics (B = 1.350, OR = 3.859; 95%CI 1.147–12.982; *p* < 0.05) and advanced training in old age psychiatry (B = 1.256, OR = 3.512; 95%CI 1.150–10.726; *p* < 0.05) had significant influences on nurses’ use of validated scales for assessing depressive symptoms. In contrast, Table 5 presents the predictors for fall risk assessments, highlighting that NH’s specialising in geriatrics (B = 2.212, OR = 9.134; 95%CI 2.085–40.006; *p* < 0.05) and advanced training in old age psychiatry (B = 1.122, OR = 3.071; 95%CI 1.212–7.782; *p* < 0.05) were the most significant predictors of nurses using validated scales for fall risk assessment. The Bs and corresponding ORs suggested that advanced training in geriatrics (B = 0.828, OR = 2.288; 95%CI 0.904–5.788; *p* = 0.081) increased the likelihood of nurses using a validated scale, although it did not reach the level of statistical significance.

Adjusting the model for age and sex improved its fit, with log-likelihood values ranging from 109.987 to 107.721, and the predictivity of using a validated scale for assessing depression, as measured by Nagelkerke’s R^2^, ranged from 0.217 to 0.242. Similarly, when adjusting the model for age and sex, the use of a validated scale for assessing fall risk showed an improved fit, with log-likelihood values from 138.150 to 131.707 and predictivity (Nagelkerke’s R^2^) ranging from 0.183 to 0.246.

### 3.8. Participants Perceptions of the Relationship Between Depressive Symptoms and Falls

Based on their experience and clinical practice, 62 (53.4%) nurses identified depression as a risk factor for falls, while 113 (97.4%) cited sedative medications and 100 (86.2%) mentioned dementia. Visual impairment was noted by 87 (75.0%) nurses, 84 (72.4%) recognised stroke as a risk factor and 78 (67.2%) noted advanced old age. Other risk factors mentioned included urinary incontinence, identified by 78 (67.2%) nurses, and antidepressant use, identified by 65 (56.0%). In contrast, a low BMI was seen as relevant by 59 (50.9%) respondents, 40 (34.5%) identified walking aids, and 32 (27.6%) noted a high BMI as a risk factor. Respiratory diseases were mentioned by 34 (29.3%) nurses and cardiac diseases by 50 (43.1%).

Most nurses (66, 56.9%) agreed with the hypothesis that NH residents with depressive symptoms were at a greater risk of falls, with 43 (37.1%) tending to agree, 12 (10.3%) agreeing and 11 (9.5%) totally agreeing. Thirty-five (43.1%) respondents were neutral, disagreed or strongly disagreed (see Table 6 for details).

## 4. Discussion

This study delved into the reported clinical practices and perceptions of nurses seeking to identify depression and fall risk among NH residents in the French-speaking canton of Vaud in Switzerland. More than half of the participating nurses reported using validated scales for assessing depressive symptoms and fall risk. They also highlighted practices such as clinical interviews with residents and families and multidisciplinary discussions, particularly with experts like physiotherapists or old age psychiatry specialists. Regarding their perceptions of peers’ practices, almost half of the nurses believed that their colleagues frequently, very often or always used validated scales to assess depressive symptoms and fall risk. Advanced training in geriatrics or old age psychiatry was associated with the increased use of validated scales for assessing depressive symptoms and fall risk. Finally, over half identified depression as a fall risk factor and believed that depressive symptoms increased fall risk; others were neutral or disagreed.

Our main findings highlighted that nurses relied on their personal experiences and intuition to identify mood changes, and they used histories of falls and gait pattern observations to assess fall risk. As the first survey in this region, it provided crucial data on clinical practices, which helped to fulfil our research aims and provided us with a deeper understanding of the strengths and weaknesses of nurses’ daily practices for detecting depression and falls in NHs. The study also scrutinised their abilities in light of current evidence-based clinical guidelines on depression and fall risk assessments in NHs.

The sample’s sociodemographic characteristics were consistent with those of a recent national study on nursing professionals [26]. Surprisingly, despite directives from the canton’s public health directorate encouraging NHs to employ nursing staff with advanced training in geriatrics or old age psychiatry, and mandating this for head nurses, our sample only included a small proportion of nurses meeting this requirement. However, this finding was consistent with existing studies by Davison et al. (2009) and Grundberg et al. (2016), which reported that NH nurses often lacked the advanced training needed to manage mental health disorders in older adults [27,28].

### 4.1. Clinical Practices for Assessing Depression and Fall Risk

The majority of the respondents reported that they primarily assessed mood disorders and fall risk through observation. They also collected health and illness histories directly from NH residents and their families. In alignment with optimised clinical practice in general, an optimal assessment of depression requires consideration of the individual and the unique aspects of their case [29,30,31]. However, evidence-based guidelines recommend that nurses’ routine clinical practices in the detection of depression should be completed with the use of appropriate tools [32,33]. Our findings showed that a significant fraction of NH nurses failed to systematically use validated scales for assessing depression; they also believed that their peers failed to do so. This may be explained by Switzerland’s lack of national and regional guidelines on the management of mood disorders in NHs. The variability in the perceived use of these scales further highlighted the need for improved training and standardisation in clinical practice. Ensuring that every nurse has the necessary skills to use validated assessment tools could improve the accurate identification of depression and fall risk, thereby improving the effectiveness of care plans. Assessments should be made far more routinely, as evidence points to the systematic use of validated scales improving the accuracy of detecting these conditions, leading to better NH resident outcomes and fewer adverse events [34,35]. The 15-item Geriatric Depression Scale was the most commonly used tool for detecting depression. This scale and its shorter versions are well validated for both major and minor depression [36]. The 15-item Geriatric Depression Scale is quickly completed and has good sensitivity, making it appropriate for depression screening among NH residents [37]. Surprisingly, we discovered a notable difference between younger nurses and their older colleagues, with older, more experienced nurses using validated scales to assess depression more frequently. This emphasised the need for a structural and organisational culture that supports routine assessments based on evidence in addition to each nurse’s personal knowledge, experience and training. Many respondents reported that they collaborated closely with a mobile team of old age psychiatry experts to ensure the correct identification of depression [34]. Consistent with evidence-based guidelines, this collaboration is supported by the canton of Vaud’s public health services and forms another part of nurses’ roles as coordinators, requiring advanced communication skills and clinical knowledge to ensure purposeful, timely assessments [30]. Multidisciplinary team discussions regarding falls and depression emphasise care coordination between nurses, physiotherapists, family physicians and mobile teams of experts in old age psychiatry [38].

The practice of assessing fall risk, as reported by the vast majority of respondents, was partially consistent with existing clinical guidelines that recognise that a recent history of falls is a strong predictor of future falls [39,40]. This underscores the importance of consistently encouraging nurses to identify NH residents’ fall histories. Since no consensus on the best assessment tool appears to have been reached, clinical utility, feasibility for individual clinicians and acceptability for patients often guide which tool is selected [41,42] and probably explain our respondents’ diverse choices. Our comparative, correlation and multivariate logistic regression analyses between nurses with more or less advanced training and their use of validated scales to assess depressive symptoms and fall risk suggest there may be a gap in current training programmes and, therefore, a potential for improvement.

Our respondents predominantly identified dementia, the use of sedatives, visual impairment and advanced age as the significant risk factors for falls, corroborating a recent study exploring nurses’ perceptions of fall risk factors in Saudi Arabia [43]. The fact that these factors are more commonly identified as fall risks than depression may explain the suboptimal use of validated scales to assess these issues concurrently. Encouragingly, more than half of our respondents identified depression as a risk factor for falls, indicating a broad recognition of its significant role in NH residents’ falls. Similarly, a comparable number of participants ‘tended to agree’ that residents with depression were at a greater risk of falling. Although these results indicate that nurses recognise the positive relationship between depression and falls, in line with the recent literature [20,44], the acknowledgement of thus link deserves to be strengthened. Indeed, recent studies support the idea that identifying specific risk factors and implementing targeted interventions for depressive symptoms can lead to additional reductions in fall risk [44,45].

### 4.2. Propositions for Clinical Practice

NH residents with multiple chronic health conditions require a comprehensive and structured evaluation process to develop resident-centred care plans and ongoing follow-up. The complex care needs of these older adults requires advanced practice nurses with the necessary clinical aptitudes and a holistic, person-centred care approach that can draw together all the varied aspects of specialist care [21]. Nurses can lead and implement this assessment process, given their role within multidisciplinary care teams and their daily interactions with NH residents. Interdisciplinary collaboration is also essential to the effectiveness of these assessments and to developing action plans. Encouraging teamwork between nurses, physicians, mental healthcare professionals and allied caregivers ensures a holistic approach to NH resident care. Regular interdisciplinary meetings can facilitate the discussion of assessment findings and the development of comprehensive care plans tailored to individual residents’ needs [46]. Finally, involving NH residents and their families in comprehensive geriatric assessment processes improves their practical relevance and quality. Emphasising to them the importance of using validated scales to detect depressive symptoms and fall risk can increase their engagement and willingness to participate actively in assessments and care planning processes [11,47,48]. Such collaboration helps create a supportive environment that promotes a better quality of life for residents [49].

The results of our multivariate logistic regressions highlighted the significant role of advanced training in geriatrics and old age psychiatry, showing them to be determinant predictors of nurses’ use of validated scales. These findings underscored the need for reinforcing the canton of Vaud’s public health policies to promote advanced nursing training in geriatrics and old age psychiatry, ensuring that healthcare professionals are well equipped to effectively utilise validated, geriatric-specific scales for identifying health issues such as depressive symptoms and fall risk. Mitchell et al. (2010) demonstrated that nurses who had undergone relevant training programmes showed significantly better knowledge about depressive disorders and improved detection skills [32]. Furthermore, supervision by professional experts in depression, with whom nurses can discuss the challenges and benefits of using screening scales, could reinforce their ongoing learning processes and should be encouraged to foster a culture of continuous improvement and lifelong learning. Small group discussions, case study exploration, professional reading and interactive learning activities could effectively meet mental health training needs [50]. Future research should explore the nuances of how advanced training in old age psychiatry affects depression and fall risk assessments. It would also be valuable to investigate whether integrating scientific knowledge about the issue of depression among NH residents and the importance of evaluating this to provide specific mental care in geriatrics and old age psychiatry could yield more substantial benefits in clinical practice.

The significant predictive results shown in our regression analysis, regarding how NHs specialising in geriatrics and NHs specialising in both geriatrics and old age psychiatry have a positive effect on nurses’ likelihood of using validated assessment tools, suggested that nurses recognised the importance of using those tools. A prior history of falls is widely recognised as a strong predictor of future falls, and a reliance on this factor—even if it was already reported by the majority of participating nurses—should be further encouraged. Predicting falls among older adults, especially those without a recorded history of them, remains nonetheless challenging [51]. It is essential to emphasise the importance of using validated tools to conduct fall risk assessments, as they provide detailed insights into specific risk factors and help guide appropriate interventions [51]. These practices should be promoted and implemented in all NHs, regardless of their clinical speciality. Incorporating considerations of fall risk factors related to mental health (e.g., depressive symptoms and dementia) in nurses’ training and NHs’ organisational planning is equally essential, given that these factors have such a significant impact on overall fall risk [39,51,52].

Examining specific managerial and organisational characteristics, such as allocating more trained human resources to better address NH residents’ mental health needs, could provide deeper insights into how to enhance assessment practices in NHs.

### 4.3. Limitations

The survey’s self-reporting questionnaire was designed specifically for the present study, so its validity could not be assessed by correlating its scores and results with a similar instrument. Self-reporting questionnaires play a central role in assessing clinical practice, but they can hinder the production of reliable answers if nurses report what they think is expected of them rather than their real clinical practices or perspectives [53]. Respondents may not answer truthfully about sensitive issues because of a social desirability bias. Although we conducted pilot tests, another potential problem is how clear or understandable items were for the NH nurses. Moreover, highly structured questionnaires can sometimes induce participants to answer in ways that do not match their true views. Finally, we hypothesise the presence of floor or ceiling effects sometimes seen in ad hoc questionnaires.

Finally, our use of a convenience sample introduced limitations related to generalisability. Since participants were selected based on their accessibility and willingness to participate, the sample may not fully represent the broader population of NH nurses, potentially limiting the applicability of our findings to other contexts. Convenience sampling also carries a higher risk of selection bias, as participants might share common characteristics or experiences that are not representative of all the nurses working in NHs. Although our exploratory study provides valuable preliminary insights, future research should address these limitations using more rigorous sampling methods to enhance the robustness and external validity of the findings.

## 5. Conclusions

Nurses can make a vital contribution to identifying depressive symptoms and fall risk among nursing home residents every time they play a leading role in using existing structured assessments for daily use based on the best available evidence. Our study found that the reported local clinical practices were partially consistent with best practices. Indeed, nursing respondents mentioned some encouraging findings, with the potential for them to make a greater and more effective contribution to depression care and fall prevention in the future. The respondents recognised the relationship between depressive symptoms and falls. The importance of recognising depression among NH residents and its relationship with falls cannot be overstated, and nurses have a valuable role to play in addressing these issues. In our study, we found a relationship between participating nurses’ advanced training and their reported use of validated scales to assess depression and fall risk. The development of skills for recognising depressive symptoms and fall risk among NH nurses must be a priority, and further commitments to implementing best practice guidelines are required. Adapting international and national recommendations, whether for depression or falls, involves reviewing global standards, understanding local challenges and customising practices to ensure they are both relevant and effective in specific NH settings. Support from policymakers is essential. Additionally, NH managers, especially head nurses, should advocate for the implementation of the comprehensive geriatric assessment framework and ensure that the Nursing Process approach is incorporated into every institution’s nursing care practices. To fully address these critical issues, further research is needed to explore and enhance nurses’ practices for the assessment and management of depressive symptoms and fall risk.

## Figures and Tables

**Table 1:** Sociodemographic and professional characteristics of the study sample (n = 116).

Sociodemographic and Professional Characteristics	n (%)	Mean (SD)	Min–Max
Sex			
Women/men	99 (85.3)/17 (17.7)		
Age in years		44.59 (11.3)	25–65
<35	31 (26.7)		
35–44	28 (24.1)		
45–54	24 (20.7)		
≥55	33 (28.4)		
Professional experience in years		18.4 (10.5)	1–43
Professional NH experience in years		13.1 (9.2)	1–40
Advanced training in geriatrics			
Yes/No	44 (37.9)/72 (62.1)		
Advanced training in old age psychiatry			
Yes/No	51 (44.0)/65 (56.0)		

**Table 2:** Nurses’ opinions about the frequency of use of validated assessment scales for depressive symptoms and fall risk by their peers (n = 116).

	Never n (%)	Rarely n (%)	Occasionally n (%)	Sometimes n (%)	Frequently n (%)	Very Often n (%)	Always n (%)	Mean (SD)	Median (IQR 1–3)
Depressive symptoms assessment using a validated scale	8 (6.9)	26 (22.4)	21 (18.1)	17 (14.7)	21 (18.1)	14 (12.1)	9 (7.8)	3.82 (1.7)	4 (3.0)
Falls risk assessment using a validated scale	14 (12.1)	25 (21.6)	5 (4.3)	18 (15.5)	27 (23.3)	15 (12.9)	12 (10.3)	3.97 (1.9)	4 (3.0)

**Table 3:** Correlation scores between having advanced training in geriatrics or old age psychiatry and the use of validated scales for assessing depressive symptoms and fall risk.

	Depressive Symptoms Assessment Using a Validated Scale Rs (95%CI)	Fall Risk Assessment Using a Validated Scale Rs (95%CI)
Advanced training in geriatrics	0.275 ** (0.092–0.440)	0.185 * (−0.003–0.360)
Advanced training in old age psychiatry	0.256 ** (0.072–0.423)	0.171 (−0.018–0.347)

Note: * *p* < 0.05; ** *p* < 0.01.

**Table 4:** Multivariate logistic regression predicting nurses’ use of validated scales for depressive symptoms assessment, adjusted for age and sex (n = 116).

Variables	B ^a^	Std. Error	Wald ^b^	Df ^c^	Sig. ^d^	Exp(B) ^e^	95% Confidence Interval for Exp(B)	
							Lower Limit	Upper Limit
Constant	−0.913	1.895	0.232	1	0.630	0.401		
NHs specialising in geriatrics	0.642	0.814	0.621	1	0.431	1.900	0.385	9.373
NHs specialising in geriatrics and old age psychiatry	−0.080	0.636	0.016	1	0.900	0.923	0.265	3.213
Professional experience in years	−0.057	0.043	1.730	1	0.188	0.945	0.868	1.028
Professional NH experience in years	0.007	0.036	0.037	1	0.848	1.007	0.938	1.081
Advanced training in geriatrics	1.350	0.619	4.758	1	0.029	3.859	1.147	12.982
Advanced training in old age psychiatry	1.256	0.570	4.862	1	0.027	3.512	1.150	10.726
Age in years	0.059	0.044	1.843	1	0.175	1.061	0.947	1.155
Sex	−0.253	0.738	0.120	1	0.729	0.777	0.187	3.234

Note. ^a^ Estimated multinomial logistic regression coefficients for the models. ^b^ The Wald chi-square test tests the null hypothesis that the estimate equals 0. ^c^ Degrees of freedom for each variable included in the model. ^d^ *p*-values. ^e^ Odds ratios for the predictors.

**Table 5:** Multivariate logistic regression predicting nurses’ use of validated scales for fall risk assessment, adjusted for age and sex (n = 116).

Variables	B ^a^	Std. Error	Wald ^b^	Df ^c^	Sig. ^d^	Exp(B) ^e^	95% Confidence Interval for Exp(B)	
							Lower Limit	Upper Limit
Constant	2.144	1.730	1.535	1	0.215	8.533		
NHs specialising in geriatrics	2.212	0.754	8.615	1	0.003	9.134	2.085	40.006
NHs specialising in geriatrics and old age psychiatry	1.087	0.573	3.592	1	0.058	2.964	0.964	9.121
Professional experience in years	0.017	0.034	0.265	1	0.607	1.017	0.953	1.086
Professional NH experience in years	−0.042	0.031	1.792	1	0.181	0.959	0.902	1.020
Advanced training in geriatrics	0.828	0.474	3.052	1	0.081	2.288	0.904	5.788
Advanced training in old age psychiatry	1.122	0.474	5.596	1	0.018	3.071	1.212	7.782
Age in years	−0.001	0.032	0.001	1	0.976	0.999	0.938	1.064
Sex	−1.736	0.755	5.281	1	0.022	0.176	0.040	0.775

Note. ^a^ Estimated multinomial logistic regression coefficients for the models. ^b^ The Wald chi-square test tests the null hypothesis that the estimate equals 0. ^c^ Degrees of freedom for each variable included in the model. ^d^ *p*-values. ^e^ Odds ratios for the predictors.

**Table 6:** Nurse respondents’ opinions about the relationship between depressive symptoms and falls among NH residents.

	I Totally Disagree, n (%)	I Do Not Agree, n (%)	I Tend to Disagree, n (%)	I Neither Agree or Disagree, n (%)	I Tend to Agree, n (%)	I Agree, n (%)	I Totally Agree, n (%)	Mean (SD)	Median (IQR 1–3)
Older adults with symptoms of depression are at a greater risk of falls.	3 (2.6)	13 (11.2)	15 (12.9)	19 (16.4)	43 (37.1)	11 (9.5)	12 (10.3)	4.44 (1.5)	5 (2.0)

## Data Availability

Requests to access these datasets should be sent to alcinaqueiros@hotmail.com.

## Supplementary Materials

The following supporting information can be downloaded at https://www.mdpi.com/article/10.3390/geriatrics9060158/s1, Supplementary file S1. Survey “Nurses’ Clinical Practice in Nursing Homes: Depressive Symptoms and Fall Risk Assessment”. Supplementary file S2. Comparison between NHs’ principal clinical speciality, nurses’ sociodemographic and professional characteristics, and validated-scale-based clinical practices for assessing depressive symptoms and fall risk.
