# Supplementary material for: Nurses’ Clinical Practice in Nursing Homes: Depressive Symptoms and Fall Risk Assessment
Source: Geriatrics (Basel). 2024 Dec 9;9(6):158. doi: 10.3390/geriatrics9060158 (PMC11727913; doi:10.3390/geriatrics9060158)
Supplement: Supplementary file 1 [file geriatrics-09-00158-s001.zip › Supplementary file S1_Nurses’ Clinical Practice in Nursing Homes- Depressive Symptoms and Falls Risk Assessment_Survey.pdf]

Nurses' Clinical Practice in Nursing Homes: Depressive Symptoms and Falls Risk Assessment

Alcina Matos Queirós [a, b], Armin von Gunten [c], Maria Manuela Martins [a], Henk Verloo [d]
[a] Institute of Biomedical Sciences Abel Salazar, University of Porto, Porto, Portugal; [b] Department of Health and Social Welfare, Lausanne, Switzerland; [c] Service of Old Age Psychiatry, Lausanne University Hospital and University of Lausanne, Prilly, Switzerland; [d] School of Nursing Sciences, University of Applied Sciences and Arts Western Switzerland, Sion, Switzerland.

Supplementary file S1 - Survey "Nurses' Clinical Practice in Nursing Homes: Depressive Symptoms and Falls Risk Assessment"

Dear Madame, Dear Sir,

The nursing homes (NHs) in the canton of Vaud are home to more than 6,000 older adults and provide them with care in a safe environment that respects their values and preferences. The health of NH residents is a central concern of the nursing profession.

We would like to invite you to participate in our survey by completing the attached questionnaire. It forms a part of the Doctoral Studies Programme in Nursing Sciences being followed by Ms Alcina Maria Matos Queirós of the Abel Salazar Institute of Biomedical Sciences in Porto, Portugal, under the supervision of Professor Henk Verloo and Professor Maria Manuela Martins. The study's objective is to understand better the clinical practices used in the canton's NHs.

We know that your time is a precious commodity, and we thank you in advance for your participation, as it will provide us with a better understanding of the clinical practices you employ for the early detection and treatment management of depression, depressive symptoms and falls among older adults living in NHs.

We would be extremely grateful if you could take the time to complete the questionnaire below by 1 March 2024. This should require about 15 minutes of your time. A stamped, addressed envelope will also be sent to your nursing home if you wish to respond using the paper version of the questionnaire.

Please reply to the questionnaire only once and based on your clinical practices and professional experiences in NHs. There are no right or wrong answers. An information sheet about the research study is attached in the annexe.

We remain at your disposal should you have any questions regarding the questionnaire or the study. Yours sincerely, and on behalf of the research team,

Alcina Maria Matos Queirós, Doctoral Candidate
Henk Verloo, Thesis Supervisor

1. Statement of informed consent

Before completing this questionnaire, please carefully read the document attached as an annexe to the questionnaire: "Detailed information for nurses, ward head nurses and nursing home head nurses in the canton of Vaud's nursing homes."
I hereby declare that I have been duly informed, by the lead investigator, about the objectives of this study on Clinical nursing practices for the management of symptoms of depression and the risk of falls among older adults living in nursing homes.

- I received satisfactory responses to the questions I asked in relation to my participation in this study. I understand the contents of the information sheet that was given to me concerning the above-mentioned study.
- I am participating in this research study voluntarily. I can, at any moment and without having to justify my actions, revoke my consent to participate in this study without this having any unfavourable repercussions on my future professional activities.

☐ Yes

2. What is your nursing home's principal clinical speciality? (More than one response possible)

○ Geriatrics
○ Old age psychiatry
○ Geriatrics and old age psychiatry
○ Other: ...

Nurses' Clinical Practice in Nursing Homes: Depressive Symptoms and Falls Risk Assessment

Alcina Matos Queirós [a, b], Armin von Gunten [c], Maria Manuela Martins [a], Henk Verloo [d]
[a] Institute of Biomedical Sciences Abel Salazar, University of Porto, Porto, Portugal; [b] Department of Health and Social Welfare, Lausanne, Switzerland; [c] Service of Old Age Psychiatry, Lausanne University Hospital and University of Lausanne, Prilly, Switzerland; [d] School of Nursing Sciences, University of Applied Sciences and Arts Western Switzerland, Sion, Switzerland.

3. In your experience, do registered nurses perform evaluations of the symptoms of depression using a validated scale? (Please tick one response only)

- ○ Never
- ○ Rarely
- ○ Occasionally
- ○ Sometimes
- ○ Often
- ○ Very often
- ○ Always

4. In your experience, do the registered nurses perform evaluations of the risk of falls using a validated scale? (Please tick one response only)

- ○ Never
- ○ Rarely
- ○ Occasionally
- ○ Sometimes
- ○ Often
- ○ Very often
- ○ Always

5. In your clinical practice, when you are evaluating a nursing home resident's symptoms of depression, which of the following methods do you use? (More than one response possible; please tick those responses corresponding to your practice)

- ☐ Observations of the patient's mood
- ☐ Clinical nursing reasoning based on experience and intuition
- ☐ Determinations following diagnosis by a physician
- ☐ Clinical nursing reasoning and the use of a validated evaluation scale for depression
- ☐ Other: ...

6. In your clinical practice, which validated clinical scales do you use for the evaluation of symptoms of depression? (More than one response possible; please tick those responses corresponding to your practice)

- ☐ None
- ☐ The Geriatric Depression Scale (4 questions)
- ☐ The Geriatric Depression Scale (15 questions)
- ☐ The Geriatric Depression Scale (30 questions)
- ☐ The Cornell Scale for Depression in Dementia
- ☐ Other: ...

Nurses' Clinical Practice in Nursing Homes: Depressive Symptoms and Falls Risk Assessment

Alcina Matos Queirós [a, b], Armin von Gunten [c], Maria Manuela Martins [a], Henk Verloo [d]

[a] Institute of Biomedical Sciences Abel Salazar, University of Porto, Porto, Portugal; [b] Department of Health and Social Welfare, Lausanne, Switzerland; [c] Service of Old Age Psychiatry, Lausanne University Hospital and University of Lausanne, Prilly, Switzerland; [d] School of Nursing Sciences, University of Applied Sciences and Arts Western Switzerland, Sion, Switzerland.

7. Please describe any other potential clinical practices that complement those noted above and that you might use to evaluate the symptoms of depression.

☐

8. In your clinical practice, when you are evaluating a nursing home resident's risk of falling, which of the following methods do you use? (More than one response possible; please tick those responses corresponding to your practice)

- ☐ Observations of the patient's walking
- ☐ Determinations following diagnosis by a physician
- ☐ The resident's history of falls in the past 9 months
- ☐ Clinical nursing reasoning based on experience and intuition
- ☐ Clinical nursing reasoning and the use of a validated evaluation scale for the risk of falls
- ☐ Other: …

9. In your clinical practice, which validated clinical scales do you use to evaluate the risk of falls? (More than one response possible; please tick those responses corresponding to your practice)

- ☐ None
- ☐ The STRATIFY Scale
- ☐ The Morse Fall Scale (MFS)
- ☐ The Timed Up-and-Go Test (TUG)
- ☐ The 6 Metre Walk Test (6MWT) at a comfortable speed
- ☐ A Heel-to-Toe-type walking test
- ☐ Other: ...

10. Please describe any other potential clinical practices that complement those noted above and that you might use to evaluate the risk of falls. (Optional response).

☐

11. Based on your clinical practice and experience, which of the following would you consider to be risk factors for falls among older adults living in NHs? (More than one response possible)

- ☐ Depression
- ☐ High BMI
- ☐ Urinary incontinence
- ☐ Low BMI
- ☐ Use of a rollator
- ☐ Use of sedative medication
- ☐ Use of antidepressant medication
- ☐ Dementia
- ☐ Use of glasses
- ☐ Visual impairment

Alcina Matos Queirós [a, b], Armin von Gunten [c], Maria Manuela Martins [a], Henk Verloo [d]
[a] Institute of Biomedical Sciences Abel Salazar, University of Porto, Porto, Portugal; [b] Department of Health and Social Welfare, Lausanne, Switzerland; [c] Service of Old Age Psychiatry, Lausanne University Hospital and University of Lausanne, Prilly, Switzerland; [d] School of Nursing Sciences, University of Applied Sciences and Arts Western Switzerland, Sion, Switzerland.

☐ Stroke

☐ Respiratory disease

☐ Cardiac disease

☐ Advanced age

☐ Other: ...

12. Older adults living in an NH and presenting with symptoms of depression are at a greater risk of falls. (Please tick one response only)

○ I totally disagree

○ I do not agree

○ I tend to disagree

○ I neither agree nor disagree

○ I tend to agree

○ I agree

○ I totally agree

13. In which year did you begin working as a nurse?

[ ]

14. In which year did you begin working as a nurse in an NH?

[ ]

15. Have you had any advanced training in geriatrics?

○ Yes

○ No

16. If you answered 'Yes' to the last item, please note the name of the advanced training programme in geriatrics that you followed. (Optional response)

[ ]

17. Have you had any advanced training in old age psychiatry?

○ Yes

○ No

18. If you answered 'Yes' to the last item, please note the name of the advanced training programme in old age psychiatry that you followed. (Optional response)

[ ]

19. In which year were you born?

[ ]

Nurses' Clinical Practice in Nursing Homes: Depressive Symptoms and Falls Risk Assessment

Alcina Matos Queirós [a, b], Armin von Gunten [c], Maria Manuela Martins [a], Henk Verloo [d]
[a] Institute of Biomedical Sciences Abel Salazar, University of Porto, Porto, Portugal; [b] Department of Health and Social Welfare, Lausanne, Switzerland; [c] Service of Old Age Psychiatry, Lausanne University Hospital and University of Lausanne, Prilly, Switzerland; [d] School of Nursing Sciences, University of Applied Sciences and Arts Western Switzerland, Sion, Switzerland.

20. Sex

- ○ Male
- ○ Female

21. You have now finished the main part of the questionnaire. Please use the box below if you would like to add any comments on the subject of depression among NH residents.

Thank you for the time you have taken to respond to this questionnaire. Your contribution will be a valuable aid in our efforts to reinforce nursing practices and thus continue to ensure the quality of care provided to the older adults living in the canton of Vaud's nursing homes.
