# Supplementary material for: Nurses’ Clinical Practice in Nursing Homes: Depressive Symptoms and Fall Risk Assessment
Source: Geriatrics (Basel). 2024 Dec 9;9(6):158. doi: 10.3390/geriatrics9060158 (PMC11727913; doi:10.3390/geriatrics9060158)
Supplement: Supplementary file 1 [file geriatrics-09-00158-s001.zip › Supplementary file S2_Nurses’ Clinical Practice in Nursing Homes- Depressive Symptoms and Falls Risk Assessment.pdf]

Nurses' Clinical Practice in Nursing Homes: Depressive Symptoms and Falls Risk Assessment

Alcina Matos Queirós [a, b], Armin von Gunten [c], Maria Manuela Martins [a], Henk Verloo [d]
[a] Institute of Biomedical Sciences Abel Salazar, University of Porto, Porto, Portugal; [b] Department of Health and Social Welfare, Lausanne, Switzerland; [c] Service of Old Age Psychiatry, Lausanne University Hospital and University of Lausanne, Prilly, Switzerland; [d] School of Nursing Sciences, University of Applied Sciences and Arts Western Switzerland, Sion, Switzerland.

Supplementary file S2 - Comparison between NHs' principal clinical speciality, nurses' sociodemographic and professional characteristics, and validated-scale-based clinical practices for assessing depressive symptoms and fall risk

Table S1a. Comparison between NHs' principal clinical speciality, nurses' sociodemographic and professional characteristics, and validated-scale-based clinical practices for assessing depressive symptoms.

Sociodemographic and professional characteristics	Total n (%)	Depressive symptoms assessment using a validated scale n (%)	Depressive symptoms assessment not using a validated scale n (%)	*p*-values
NH's principal clinical speciality				< 0.01 **
Geriatrics	25 (21.6)	21 (18.1)	4 (3.4)	
Geriatrics and old age psychiatry	70 (60.3)	51 (44.0)	19 (16.4)	
Old age psychiatry	21 (18.1)	16 (13.8)	5 (4.3)	
Age in years				< 0.01 **
< 35	31 (26.7)	17 (14.7)	14 (12.1)	
35–44	28 (24.1)	24 (20.7)	4 (3.4)	
45–54	24 (20.7)	19 (16.4)	5 (4.3)	
≥ 55	33 (28.4)	28 (24.1)	5 (4.3)	
Nurses' years of professional experience				< 0.01 *
< 18	48 (41.4)	40 (34.5)	8 (6.9)	
≥ 18	68 (58.6)	48 (41.4)	48 (41.4)	
Professional NH experience in years				
< 13	57 (49.1)	40 (34.5)	17 (14.7)	
≥ 13	59 (50.9)	48 (41.4)	11 (9.5)	
Advanced training in geriatrics				< 0.01 *
Yes / No	44 (37.9) / 72 (62.1)	40 (3.4) / 48 (41.4)	4 (3.4) / 24 (20.7)	
Advanced training in old age psychiatry				< 0.01 *
Yes / No	51 (44) / 65 (56.0)	45 (38.8) / 43 (37.1)	6 (5.2) / 22 (19.0)	

*Chi-squared **Cramér's V

Table S1b. Comparison between NHs' principal clinical speciality, nurses' sociodemographic and professional characteristics, and validated-scale-based clinical practices for assessing falls risk.

Sociodemographic and professional characteristics	Total n (%)	Falls risk assessment using a validated scale n (%)	Falls risk assessment not using a validated scale n (%)	*p*-values
NH's principal clinical speciality				< 0.01 **
Geriatrics	25 (21.6)	20 (17.2)	5 (4.3)	
Geriatrics and old age psychiatry	70 (60.3)	42 (36.2)	28 (24.1)	
Old age psychiatry	21 (18.1)	9 (7.8)	12 (10.3)	
Age in years				< 0.01 **
< 35	31 (26.7)	18 815.5)	13 (11.2)	
35–44	28 (24.1)	18 (15.5)	10 (8.6)	
45–54	24 (20.7)	10 (8.6)	14 (12.1)	
≥ 55	33 (28.4)	25 (21.6)	8 (6.9)	
Nurses' years of professional experience				< 0.01 *
< 18	48 (41.4)	30 (25.9)	18 (15.5)	
≥ 18	68 (58.6)	41 (35.3)	27 (23.3)	
Professional NH experience in years				
< 13	57 (49.1)	34 (29.3)	23 (19.8)	
≥ 13	59 (50.9)	37 (31.9)	22 (19.0)	
Advanced training in geriatrics				< 0.01 *
Yes / No	44 (37.9) / 72 (62.1)	32 (27.6) / 39 (33.6)	12 (10.3) / 33 (28.4)	
Advanced training in old age psychiatry				< 0.01 *
Yes / No	51 (44) / 65 (56.0)	36 (31.0) / 35 (30.2)	15 (12.9) / 30 (25.9)	

*Chi-squared **Cramér's V
